# Jackhammer Esophagus Presenting as Chest Discomfort and Dyspnea in a Patient With Undifferentiated Inflammatory Arthritis and a Positive Scl-70 Antibody Test

**DOI:** 10.7759/cureus.117601

**Published:** 2026-10-07

**Authors:** Giuliana Perini Villanueva, Alex Kokaly, Nisha Sunku, Christina W Chung

**Affiliations:** 1 Internal Medicine, Ronald Reagan UCLA (University of California, Los Angeles) Medical Center, Los Angeles, USA; 2 Medicine, David Geffen School of Medicine at UCLA (University of California, Los Angeles), Los Angeles, USA; 3 Internal Medicine, UCLA (University of California, Los Angeles) Health, Los Angeles, USA; 4 Internal Medicine, UCLA (University of California, Los Angeles) Santa Monica Medical Center, Santa Monica, USA

**Keywords:** atypical chest pain, connective tissue disease, esophageal motility disorder, gastro intestinal motility, jackhammer esophagus

## Abstract

Chest discomfort in patients with autoimmune connective tissue disease often prompts extensive cardiopulmonary evaluation, yet esophageal motility disorders remain an underrecognized etiology.

In this report, we present a case of a 34-year-old woman with Raynaud's phenomenon, undifferentiated inflammatory arthritis (ANA- and Scl-70-positive), gastroesophageal reflux disease (GERD), and small intestinal bacterial overgrowth (SIBO) who developed progressive dyspnea and chest tightness following an influenza A infection. Despite an unremarkable broad initial workup that included computed tomography (CT) pulmonary angiography, echocardiography, and pulmonary function testing, high-resolution esophageal manometry with impedance, along with supplemental testing (endoluminal functional lumen imaging probe (EndoFLIP) and barium esophagram), ultimately supported a diagnosis of jackhammer esophagus (JE), a rare hypercontractile esophageal motility disorder.

This case highlights JE as a rare and underrecognized cause of chest symptoms that should be considered when cardiopulmonary evaluation is unrevealing, particularly in patients with underlying autoimmune connective tissue disease, positive SSc serological markers, and a history of gastrointestinal disease.

## Introduction

Gastrointestinal causes of chest pain account for 10%-20% of presentations yet are often considered only after extensive cardiopulmonary evaluation [1]. Esophageal motility disorders can produce retrosternal chest pain and tightness that closely mimic cardiac or pulmonary disease [2].

Jackhammer esophagus (JE) is a rare hypercontractile motility disorder defined by greater than or equal to two swallow maneuvers with a distal contractile integral (DCI) greater than 8,000 mmHg/s/cm on high-resolution manometry (HRM). Its prevalence among patients referred for HRM is approximately 2%, with a female majority (around 65%). Dysphagia is the most common symptom, followed by classic gastroesophageal reflux disease (GERD) symptoms such as heartburn, regurgitation, and belching, as well as chest pain. Chest pain in JE can imitate cardiac-type chest pain, such as that seen in angina or myocardial infarction, and may be debilitating [2-5].

The pathogenesis is thought to involve excessive cholinergic drive with abnormal inhibition of neuromuscular function. JE has been associated with systemic autoimmune diseases, connective tissue diseases, GERD, eosinophilic esophagitis, and opioid use. Some proposed mechanisms include opioid-mediated impairment of inhibitory nitrous oxide signaling and potential effects of eosinophil-derived mediators, although causal relationships are unclear [6,7].

Some studies show that systemic sclerosis (SSc) involves the gastrointestinal tract in around 90% of patients, typically causing absent contractility or ineffective esophageal motility [8-9]. Other studies show that esophageal motility disorders are found in 58% of patients with any rheumatologic disease, including hypercontractile patterns such as JE (8% in one cohort) [10]. This case illustrates a patient with undifferentiated inflammatory arthritis and Scl-70 antibody positivity, though without a confirmed diagnosis of SSc, whose chest discomfort was ultimately diagnosed as JE after a negative cardiopulmonary workup.

## Case presentation

A 34-year-old female presented with progressive dyspnea and chest tightness described as "someone hugging (her) too tight," with onset one month prior to presentation. Her history was significant for Raynaud's phenomenon, undifferentiated inflammatory arthritis (ANA and Scl-70 positive, currently on upadacitinib and methotrexate), peripheral neuropathy, tremors/myoclonus, panniculitis, and type 2 diabetes. Of note, she had been evaluated by rheumatology in 2024 for SSc and did not meet formal classification criteria at that time. She was a former agricultural worker and non-smoker.

Her gastrointestinal history included GERD with regurgitation, mixed irritable bowel syndrome (IBS), small intestinal bacterial overgrowth (SIBO), and low fecal elastase on pancreatic enzyme replacement. Prior pulmonary workup had shown a calcified right lower lobe (RLL) nodule, which on biopsy showed fibrotic scar and was negative for malignancy. Tuberculosis (TB) and coccidioidomycosis testing were negative. Pulmonary function tests (PFTs) two months prior to presentation showed a reversible moderate obstructive defect and severely reduced maximal inspiratory pressure (MIP)/maximal expiratory pressure (MEP).

One month prior to presentation, she tested positive for influenza A that was untreated at the time. Respiratory symptoms persisted and worsened, and a few weeks after the onset of symptoms, she required daily albuterol without significant benefit. She was subsequently diagnosed with acute bronchitis at an outside clinic/urgent care and prescribed azithromycin and a prednisone taper, also without improvement. One week later, she was unable to get out of bed due to severe, worsening shortness of breath.

She presented to the Emergency Department at the recommendation of her pulmonologist. She was afebrile and hemodynamically stable. Abdominal examination was benign, and cardiopulmonary examination demonstrated clear lungs bilaterally, though air movement was diminished throughout. Immunoglobulin E (IgE), quantitative serum immunoglobulins, erythrocyte sedimentation rate (ESR), and C-reactive protein (CRP) were all within normal limits. Thyroid-stimulating hormone (TSH) was elevated at 7.0 with a normal free T4 (0.90); this finding was not addressed further or treated during the admission. Coccidioidomycosis serology was negative. All relevant results can be seen in Table 1.

**Table 1:** Laboratory trend table by hospitalization day. H: abnormally high; L: abnormally low; -: not drawn/not applicable

Lab (Reference Range)	Day 1	Day 2	Day 3	Day 4
White Blood Cell (count) (4.16-9.95 x 10³/µL)	13.21 (H)	10.08 (H)	7.34	7.28
Hemoglobin (11.6-15.2 g/dL)	12.4	10.9 (L)	10.8 (L)	10.9 (L)
Platelet Count (143-398 x 10³/µL)	186	151	161	166
Sodium (135-146 mmol/L)	141	142	139	141
Potassium (3.6-5.3 mmol/L)	3.4 (L)	3.7	3.6	3.6
Chloride (96-106 mmol/L)	110 (H)	113 (H)	109 (H)	110 (H)
Total CO2 (20-30 mmol/L)	17 (L)	19 (L)	20	20
Blood Urea Nitrogen (BUN) (7-22 mg/dL )	11	13	11	12
Creatinine (0.60-1.30 mg/dL)	0.65	0.74	0.73	0.68
Glucose (65-99 mg/dL)	118 (H)	83	94	95
Calcium (8.6-10.4 mg/dL)	8.9	8.5 (L)	8.9	8.8
Aspartate Aminotransferase (AST) (13-62 U/L)	18	13	12 (L)	19
Alanine Aminotransferase (ALT) (8-70 U/L)	17	12	13	22
Alkaline Phosphatase (37-113 U/L)	61	53	54	58
Bilirubin, Total (0.1-1.2 mg/dL)	0.3	0.2	0.3	0.2
Bilirubin, Conjugated (≤0.3 mg/dL)	-	<0.2	<0.2	<0.2
Albumin (3.9-5.0 g/dL)	4.6	3.8 (L)	3.9	3.8 (L)
Total Protein (6.1–8.2 g/dL)	7.4	6.5	6.6	6.1
High-Sensitivity Troponin I (<5 ng/L)	<4	-	-	-
Brain Natriuretic Peptide (BNP) (<100 pg/mL)	26	-	-	-
D-Dimer (<0.60 µg/mL FEU)	0.64 (H)	-	-	-
Immunoglobulin E (kIU/L)	<2	-	-	-
Thyroid-Stimulating Hormone (0.3-4.7 mcIU/mL)	-	7.0 (H)	-	-
Free Thyroxine (T4) (0.80-1.70 ng/dL)	-	0.90	-	-
Cocci IgG Enzyme Immunoassay (<0.150)	-	<0.150	-	-
Cocci IgM Enzyme Immunoassay (<0.150)	-	<0.150	-	-
Immunoglobulin G, Serum (700-1600 mg/dL)	-	900	-	-
Immunoglobulin A, Serum (76-426 mg/dL)	-	141	-	-
Immunoglobulin M, Serum (40-230 mg/dL)	-	58	-	-
C-Reactive Protein (CRP) (<0.8 mg/dL)	-	0.7	-	-
Immunoglobulin G Subclass 1 (240-1118 mg/dL)	-	-	404	-
Immunoglobulin G Subclass 2 (124-549 mg/dL)	-	-	240	-
Immunoglobulin G Subclass 3 (21-134 mg/dL)	-	-	70	-
Immunoglobulin G Subclass 4 (1-123 mg/dL)	-	-	44	-
Alpha-1-Antitrypsin (90-200 mg/dL)	-	-	124	-

Computed tomography angiography (CTA) of the chest was negative for pulmonary embolism (PE), with no acute parenchymal findings and an unchanged calcified RLL nodule. Transthoracic echocardiogram (TTE) was normal. Inpatient pulmonary function testing showed no obstructive defect (improved from prior), a mild restrictive defect, severely reduced MIP, and normal diffusion capacity. The pulmonology consultants concluded the dyspnea was likely due to extrapulmonary causes.

After excluding PE, pneumonia, heart failure, acute coronary syndrome (ACS), interstitial lung disease (ILD), and primary obstructive airway disease, the differential diagnosis shifted toward costochondritis and gastrointestinal etiologies as potential causes of her chest discomfort. Given her history of dysphagia, GERD, SIBO, and concern for SSc-spectrum disease, gastroenterology was also consulted. Esophagogastroduodenoscopy (EGD) revealed erosive gastropathy with an otherwise normal-appearing esophagus. HRM with impedance, which evaluates the pressure and strength of esophageal muscle contractions, demonstrated a hypercontractile pattern with 40% of swallows exhibiting a DCI >8,000 mmHg/cm/sec (normal <20%). Resting lower esophageal sphincter (LES) pressure was elevated at 49.9 mmHg (normal 4.8-32 mmHg), and the median integrated relaxation pressure (IRP) was markedly elevated in both the supine (23.7 mmHg; normal <15.0 mmHg) and upright (39.7 mmHg; normal <12.0 mmHg) positions, raising concern for impaired esophagogastric junction (EGJ) relaxation versus possible concomitant esophagogastric junction outflow obstruction (EGJOO). Distal latency and panesophageal pressurization were normal. The impedance component, which assesses movement of boluses by detecting changes in electrical resistance as material passes through the esophagus, demonstrated preserved liquid (90%) and viscous (100%, with partial bolus escape) bolus clearance, indicating that despite abnormal contractile patterns, esophageal transit remained intact.

Overall, the study findings and clinical symptoms were most consistent with JE, with the elevated IRP raising initial concern for concomitant EGJ outflow obstruction that was further evaluated, as discussed below. Given her spontaneously improving symptoms, stable respiratory status on room air, and initiation of proton pump inhibitor (PPI) therapy, she was reasonably discharged with planned outpatient follow-up in pulmonology, rheumatology, and gastroenterology. Manometry results were pending at the time of discharge, and the diagnosis of JE was formally established in the outpatient setting several days later (Figure 1).

**Figure 1:**
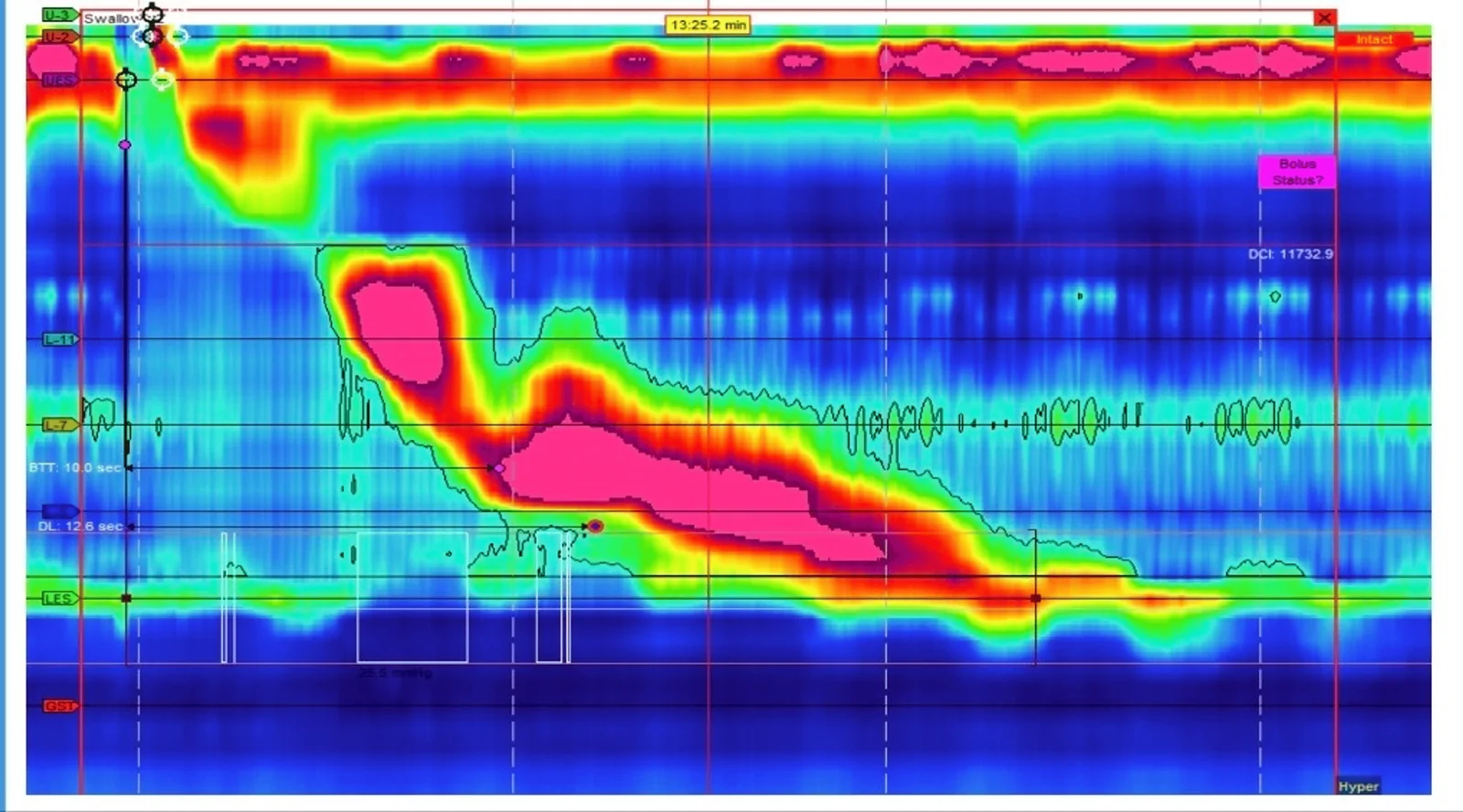
High-resolution manometry with impedance demonstrating findings consistent with jackhammer esophagus. Representative swallow showing the patient's hypercontractile esophageal response (distal contractile integral >8,000 mmHg/s/cm) with an elevated integrated relaxation pressure (IRP), initially raising concern for concomitant esophagogastric junction outflow obstruction on manometry alone. Subsequent endoluminal functional lumen imaging probe (EndoFLIP) and barium esophagram findings, together with preserved bolus clearance on impedance testing, did not support a clinically significant obstructive process, favoring a diagnosis of isolated jackhammer esophagus per Chicago Classification v4.0.

Following the diagnosis of JE, gastroenterology recommended medical management with peppermint, nitrates, or calcium channel blockers. Further evaluation with endoluminal functional lumen imaging probe (EndoFLIP) and barium esophagram was also pursued to characterize the elevated IRP noted on manometry. EndoFLIP demonstrated normal EGJ opening with a disordered contractile response, and barium esophagram was negative for achalasia, making a clinically significant concomitant EGJ outflow obstruction less likely and favoring isolated JE as the more likely diagnosis; gastroenterology continued to recommend a timed barium swallow, which has not yet been performed. The patient elected to trial peppermint therapy and is currently taking one to two peppermint Altoids sublingually 10-15 minutes before meals, though she has not reported recent updates on symptomatic improvement. Given the pathophysiologic overlap between JE and GERD, empiric PPI therapy was continued [6].

## Discussion

This case highlights JE, a rare hypercontractile motility disorder of the esophagus, as an underrecognized cause of chest discomfort in a patient with autoimmune disease. Several aspects of this case merit further discussion, including the overlap between JE and cardiopulmonary disease, the pathogenesis of hypercontractile esophageal patterns in the context of connective tissue disease, the evolving diagnostic framework for JE, the natural history of the disorder, and the current treatment landscape.

JE can produce chest pain that imitates cardiac angina, as was the case in this patient. The cause of esophageal pain in motility disorders is multifactorial, involving sustained contraction of the longitudinal muscles, reduced esophageal wall blood flow, upregulation of nociceptive receptors (e.g., vanilloid receptor 1, acid-sensitive ion channels), and hypersensitivity of spinal and supraspinal neural pathways [2,11]. In a meta-analysis of 38 studies, chest pain was present in approximately one-third of JE patients (30.7%), while dysphagia (64%) and GERD symptoms (46%) were more common [5]. The 2021 American Heart Association/American College of Cardiology (AHA/ACC) Chest Pain Guidelines recommend evaluation for gastrointestinal causes when cardiac and pulmonary etiologies are excluded [1]. In this patient, the extensive negative cardiopulmonary workup - including CTA, TTE, PFTs with normal diffusing capacity of the lungs for carbon monoxide (DLCO), and negative infectious studies - ultimately directed attention to the esophagus. It is important to note that dyspnea, this patient's most prominent presenting symptom, is not a classically described feature of JE; the condition is primarily associated with dysphagia, chest pain, and GERD-related symptoms [5]. While her chest tightness can be explained by JE, her dyspnea may have had multiple contributing causes, including the recent influenza infection, general deconditioning, or another factor not identified by the cardiopulmonary workup. Additionally, her dyspnea had largely resolved by discharge, before the manometric diagnosis and new treatment were finalized, further limiting any clear temporal link between the two. This further suggests that JE may not fully account for everything she experienced.

Notably, the Chicago Classification version 4.0 (CCv4.0) for esophageal motility disorders now requires a normal median IRP, exclusion of mechanical obstruction, and clinically relevant symptoms (dysphagia and/or non-cardiac chest pain) for a conclusive diagnosis of hypercontractile esophagus, aiming to limit overdiagnosis and enhance clinical relevance of the manometric finding [12]. This patient's chest tightness and history of dysphagia satisfy the symptomatic criterion; however, her manometry showed an elevated IRP in both supine (23.7 mmHg) and upright (39.7 mmHg) positions, and the initial manometric interpretation raised a combined diagnosis of JE with concomitant EGJOO, with the manometry report noting it was unclear how much of the IRP elevation reflected true outflow obstruction versus an artifact of LES hypercontractility itself. CCv4.0 recommends further testing such as EndoFLIP and timed barium esophagram to clarify inconclusive manometric findings. In this case, EndoFLIP demonstrated normal EGJ opening, barium esophagram was negative for achalasia, and there was a normal bolus clearance on impedance testing. These findings did not support a clinically significant obstructive process, and the final diagnosis was therefore isolated JE rather than a combined motility disorder. A timed barium swallow, which was recommended and can provide additional supportive evidence, was not performed [12].

The finding of JE in a patient with SSc-spectrum markers raises the question of whether the hypercontractile pattern represents an early or atypical manifestation of autoimmune esophageal involvement, different from the classic hypocontractile SSc esophagus. While "classic scleroderma esophagus" causes absent contractility and a reduced EGJ/LES pressure, this pattern was observed in only 33% of SSc patients in one large cohort, reaffirming the variety of esophageal motor dysfunction in SSc [9]. Hypercontractile patterns were identified in 9% (n = 26) of patients with underlying rheumatologic disease undergoing manometry, including diffuse esophageal spasm (1.4%, n = 4) and JE (8%, n = 22) [10].

The pathogenesis of esophageal dysmotility is not well-characterized. One theory proposes that the pathogenesis in SSc is a process that begins with neuropathy that then progresses to myopathy and ends in fibrosis. In the earliest stage, circulating autoantibodies disrupt cholinergic neurotransmission at the myenteric plexus, with the smooth muscle itself staying intact and the effects potentially reversible. With disease progression, myopathy and eventual fibrosis make the esophagus unresponsive [13]. Other studies have shown that autonomic nervous dysfunction has also been significantly associated with esophageal dysmotility in SSc, and autopsy studies have confirmed smooth muscle atrophy in 94% of SSc esophaguses [14-15].

The three-stage model for the pathogenesis of esophageal dysmotility raises an interesting hypothesis proposed in the literature: hypercontractile patterns such as JE have been suggested as a possible early neuropathic phase in some SSc-spectrum patients, characterized by impaired inhibitory pathways. Under this model, loss of inhibitory neural input leads to unopposed excitatory cholinergic activity, resulting in hypercontractility before eventual decline in contractile function. Supporting this hypothesis, provocative testing (additional maneuvers performed during high-resolution manometry that challenge esophageal function beyond standard swallows) has demonstrated abnormal motor inhibition in 48% of patients with JE during multiple rapid swallows, with 66% lacking contraction reserve. Contraction reserve refers to the esophagus’s ability to increase contraction strength after repeated swallows. Reduced reserve suggests impaired neural regulation and may indicate neuropathic dysfunction [16]. 

This patient had Raynaud's phenomenon and peripheral neuropathy, conditions that point to nerve and blood vessel involvement outside the esophagus, raising the question of whether a similar process could be affecting the esophagus in this case. Whether the JE is a separate, unrelated finding or connected through a shared nerve-related mechanism remains unclear. Important to note that these frameworks remain largely hypothetical in the current patient, because despite having SSc-associated serology, she does not have a confirmed SSc diagnosis.

However, esophageal motor disorders are not limited to SSc. In a cohort of 289 patients with various rheumatologic diseases who underwent HRM, 58% had an esophageal motility disorder, including achalasia (5%), EGJ outflow obstruction (20%), JE (8%), ineffective esophageal motility (28%), and distal esophageal spasm (1%) [10]. Notably, 50% of patients with a normal barium swallow had esophageal dysmotility on HRM, highlighting the superior sensitivity of manometry [10]. In a separate cohort, 61.1% of patients with underlying rheumatologic disease had esophageal motility disorders on HRM with impedance [17]. These data show the prevalence of esophageal dysmotility in rheumatologic conditions and support the recommendation that HRM should be considered in patients with rheumatic conditions presenting with esophageal symptoms, even when other testing is unremarkable.

The natural course of JE remains poorly understood. While JE is generally considered a benign disorder that rarely progresses, progression to type II achalasia (characterized by failed LES relaxation with pressure buildup throughout the esophageal body) has been documented in case reports [18]. This suggests that JE may be an early manifestation of progressive esophageal motility dysfunction. This is in line with the hypothesis mentioned above, in which SSc esophageal involvement is posited to develop along the pathway of neuropathy to myopathy to fibrosis. In SSc specifically, absent contractility is associated with a more severe clinical phenotype, including diffuse skin disease, more severe Raynaud's, reduced DLCO, and higher mortality [8]. Longitudinal follow-up in this patient will be critical to determine whether her autoimmune conditions or her hypercontractile pattern evolves over time.

Treatment of JE remains challenging due to insufficient understanding of the underlying mechanisms and limited high-quality evidence. The approach is generally stepwise. First-line management includes addressing reversible causes such as GERD and eosinophilic esophagitis; empiric PPI optimization is recommended given overlap of GERD symptoms with hypercontractile esophagus. Pharmacologic options include smooth muscle relaxants such as calcium channel blockers, nitrates, and peppermint oil, though controlled trial data are limited [6,7].

For refractory cases, peroral endoscopic myotomy (POEM), which involves targeted transection of esophageal smooth muscle fibers to reduce hypercontractility, has emerged as a minimally invasive option. A meta-analysis of nine studies including 210 patients with spastic esophageal disorders reported a pooled clinical success rate of 89.6% (95% CI 83.5-93.1) [19]. However, the 2024 American Gastroenterological Association (AGA) Clinical Practice Update recommends exhausting less invasive options before considering POEM for non-achalasia spastic motility disorders [20].

The patient's rheumatologic diagnosis remains undifferentiated, and longitudinal follow-up will determine whether she meets formal SSc classification criteria in the future. Additionally, the natural history of JE in the context of connective tissue disease is unknown, and it is uncertain whether the hypercontractile pattern will persist, resolve, or evolve into a hypocontractile pattern over time.

## Conclusions

JE is a rare but clinically significant esophageal motility disorder that can present with chest discomfort difficult to distinguish from cardiac or pulmonary disease. This case illustrates the diagnostic challenges that arise when a patient with autoimmune disease and SSc-spectrum serological markers presents with unexplained chest and respiratory symptoms and underscores the importance of maintaining a broad differential that includes esophageal etiologies. The extensive negative cardiopulmonary workup in this patient ultimately redirected attention to the esophagus, where HRM with impedance, together with EndoFLIP and barium esophagram, supported a diagnosis of isolated JE. This case highlights an important diagnostic consideration in rheumatologic populations, where esophageal symptoms may be attributed to known comorbidities such as GERD or SIBO rather than prompting dedicated evaluation for esophageal dysmotility

The pathophysiology of JE in the context of connective tissue disease remains incompletely understood. This patient's rheumatologic diagnosis remains undifferentiated, and it is not yet known whether she will meet formal SSc classification criteria. Therefore, the discussion of SSc-associated mechanisms in this report should be interpreted as hypothesis-based rather than diagnostic. With that caveat, emerging theories support a neurogenic mechanism in which impaired inhibitory neuromuscular signaling leads to unopposed cholinergic excitation and hypercontractility, and some have proposed that hypercontractile patterns may represent an early phase of esophageal involvement in SSc-spectrum disease before fibrosis and smooth muscle atrophy occur. The Chicago Classification version 4.0 has refined the diagnostic criteria for JE to require clinically concordant symptoms alongside manometric findings and to account for elevated IRP and possible concomitant EGJ outflow obstruction, as seen in this case. Additional tools such as EndoFLIP and timed barium esophagram continue to expand our ability to characterize these disorders. Treatment remains largely empiric, with PPI optimization, smooth muscle relaxants, and, in refractory cases, POEM offering meaningful symptom relief. Taken together, this case argues for earlier esophageal evaluation with endoscopy and HRM in patients with unexplained chest symptoms and rheumatologic disease, including disease that remains undifferentiated, and highlights the need for longitudinal observation of esophageal motility patterns in this population.
